# Pericardial Tuberculosis in a Non-endemic Region Presenting as a Persistent Upper Respiratory Tract Infection With Negative Serology, Bronchial Washings, and Pleural Aspirate

**DOI:** 10.7759/cureus.52227

**Published:** 2024-01-13

**Authors:** Philip Nolan, Sanya Samad, Thomas Kiernan

**Affiliations:** 1 Cardiology, Waikato Hospital, Hamilton, NZL; 2 Cardiology, University Hospital Galway, Galway, IRL; 3 Cardiology, University Hospital Limerick, Limerick, IRL; 4 Internal Medicine, Waikato Hospital, Hamilton, NZL; 5 Internal Medicine, University Hospital Limerick, Limerick, IRL

**Keywords:** case report, pericardiectomy, echocardiography, tuberculosis, cardiology, pericardial diseases, extrapulmonary tuberculosis (eptb)

## Abstract

This paper reports on the unlikely case of a 68-year-old man presenting with a non-resolving, mild lower respiratory tract infection, subsequently diagnosed with pericardial tuberculosis (TB) in the absence of TB risk factors and with negative TB serology.

Pericardial and pleural effusions were found incidentally on CT pulmonary angiogram, with a small pericardial effusion without tamponade seen on the echocardiogram. During his three-month inpatient stay, the patient was rarely very unwell, though no treatment led to clinical and biochemical resolution of symptoms. Later deterioration prompted another echocardiogram, which found a moderate-sized pericardial effusion, septal bounce, and new regional wall motion abnormalities. To avert the impending cardiac tamponade, the patient underwent pericardiectomy, which provided a tissue diagnosis of TB.

Pericardial TB is extremely uncommon, especially outside of TB endemic regions, though it is well described. This case is especially noteworthy, as serology, bronchial washings, and pleural aspirate had been negative for TB though a Quantiferon test was positive. The diagnosis was only confirmed after pericardiectomy. The patient was subsequently treated with anti-TB therapy, with a good clinical response. This case highlights diagnostic challenges and strategies for investigating and managing similar complex scenarios, particularly in non-endemic settings.

## Introduction

Pericarditis is a rare manifestation of tuberculosis [1]. It is rarer still in the absence of classic TB risk factors such as immunosuppression, homelessness, malignancy, and tumor necrosis factor-alpha (TNF-α) inhibitor use [2]. The significance of this case lies not only in the rarity of the diagnosis in non-endemic regions but also in the lack of these predisposing factors [3]. This patient did not present with symptoms typical of pericarditis, nor did he present with constitutional symptoms of tuberculosis. This patient was referred by his primary care provider (PCP) for a non-resolving respiratory tract infection. Multiple blood cultures and polymerase chain reaction (PCR) tests, sputum samples, bronchoalveolar washings, and pleural effusion fluid culture were negative for tuberculosis. Diagnosis of pericardial tuberculosis was only confirmed following pericardiectomy.

TB is uncommon in Ireland, with 315 cases and a reported incidence rate of 6.6 per 100,000 in 2018 [4]. While gross numbers are similar, the rate of TB is higher in foreign-born individuals. In 2018, there were no incidences of pericardial TB in Ireland [4].

There was considerable uncertainty in the diagnosis throughout this patient’s three-month stay in the hospital. While he was rarely clinically very unwell, biomarkers did not fall to an acceptable level for him to be deemed fit for discharge. The focus of investigations and treatment was the respiratory system, with the pericardial effusion thought to be a result of this. Up until confirmation of TB diagnosis with pericardial tissue sampling, other etiologies were considered, including radiation pericarditis from previous radiation to the prostate. Ultimately, a multidisciplinary approach, involving cardiology, geriatrics, infectious disease, cardiothoracics, and radiology, arrived at the correct diagnosis.

## Case presentation

This patient was a 68-year-old retired builder. He was referred to the University Hospital Limerick (UHL) emergency department by his primary care physician (PCP) following what was thought to be an unresolved upper respiratory tract infection. This had been treated with three courses of antibiotics with no total relief of symptoms. On admission, the patient was finishing a course of co-amoxiclav and doxycycline. His presenting symptoms were shortness of breath, productive cough, and recurrent fevers for over one month’s duration. The cough was noted to be productive of initially green sputum, which had progressed to clear sputum.

In the week prior to admission, the patient’s condition had deteriorated and he had experienced rigors, sweating, and an episode of vertigo treated with betahistine. The PCP noted his O_2_ saturation was 89% on room air with a temperature of 39°C.

The patient’s past medical history included prostate cancer treated with radiotherapy, hypertension, and hyperlipidemia. His regular medications were rosuvastatin and amlodipine, with a new prescription for betahistine. The patient was a lifelong non-smoker and consumed alcohol at weekends. He lived in a well-maintained house, had no sick contacts, and had no prior significant TB exposure.

On examination, the patient was pyrexical at 39°C, but vitals were otherwise stable. He had scattered crackles and wheezes over both lungs, the abdomen was soft with mild epigastric tenderness, and no audible murmurs. ECG showed normal sinus rhythm. Chest X-ray showed cardiomegaly and left basal consolidation. This was felt to be attributable to a posteroanterior positioning rather than pathology. Blood tests showed hyponatremia (129 mEq/L) and venous blood gas (VBG) showed respiratory alkalosis (pH: 7.456, CO_2_: 4.13 kPa). The attending physician’s impression was of a lower respiratory tract infection. The patient was started on 4.5 g intravenous (IV) piperacillin-tazobactam, 1 g paracetamol PO, and IV saline. Soon after admission, the patient developed atrial fibrillation and was started on enoxaparin.

**Figure 1:**
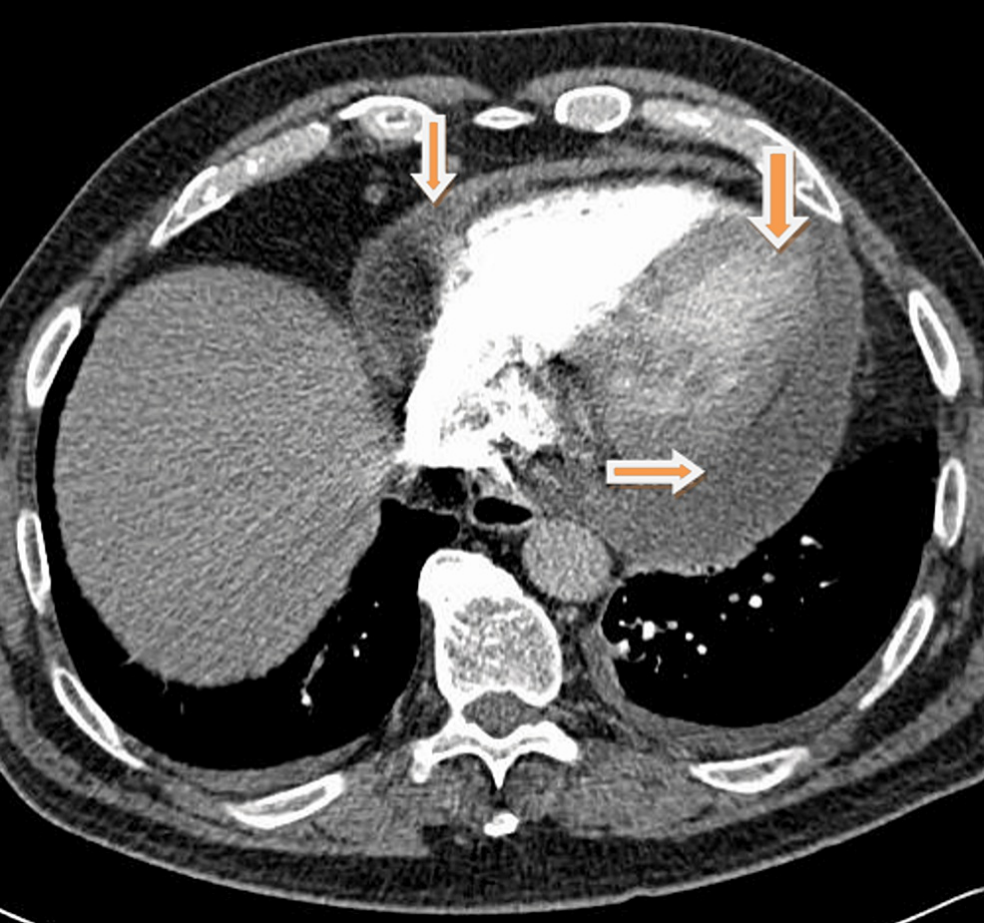
CTPA with pericardial effusion indicated CTPA: computed tomography angiography

**Figure 2:**
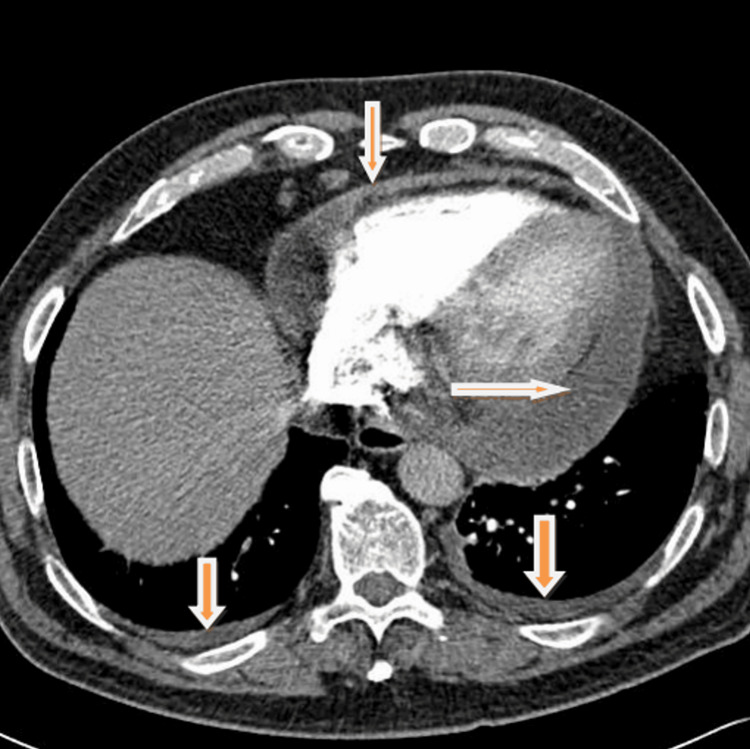
CTPA with pleural effusion and pericardial effusion shown CTPA: computed tomography angiography

The patient was booked for a computed tomography angiography (CTPA) as his D-dimer was raised (2.28 mg/L). He was clinically stable though he had widespread crepitations on auscultation of the chest. The CTPA was reported as negative for PE with a ‘large ring-enhancing pericardial effusion, measuring 3.2 cm at maximal depth’ (Figure 1), which was ‘consistent with pericarditis’, ‘reflux of contrast to the hepatic veins, consistent with right-heart dysfunction’, and a right base pleural effusion (Figure 2). Sputum and blood cultures had been reported as negative and were repeated. The pericardial effusion was felt to be due to infection, though paraneoplastic syndrome and metastases were also considered due to the patient’s history of prostate cancer. TB was included as a differential following cardiology consultation.

**Figure 3:**
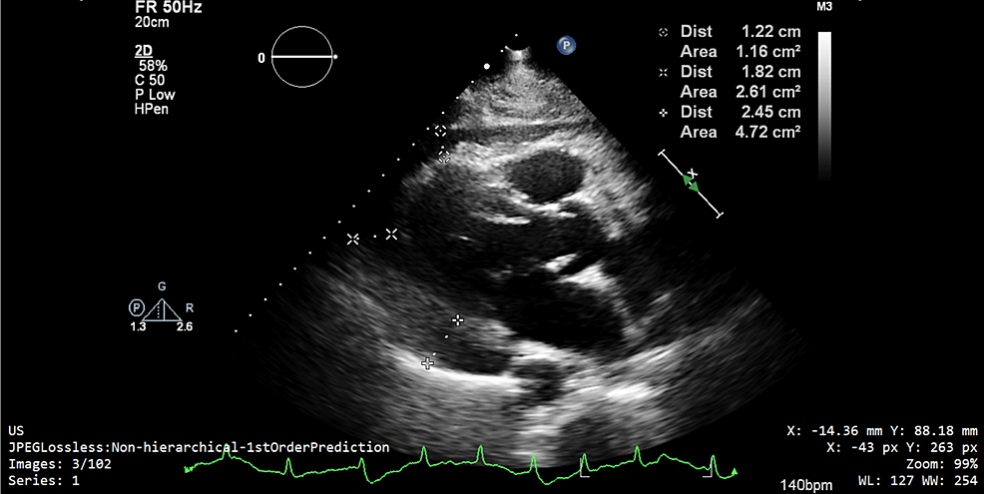
Pericardial effusion seen on echocardiogram, 2.45 cm at its largest

An echocardiogram showed preserved left ventricular function with a moderate-sized pericardial effusion, 2.4 cm at its largest (Figure 3), and no evidence of tamponade. Further workup for the cause of the pericardial effusion did not point to a clear reason. PSA was not significantly raised. CT thorax-abdomen-pelvis showed no further collections. A diagnostic tap of the pleural fluid showed straw-colored aspirate but was negative for culture growth and for acid-fast bacilli. The pleural effusion was exudative, which prompted further workup for TB or metastases. Bronchoscopy and bronchoalveolar lavage were negative for acid-fast bacilli and culture growth. Repeat blood cultures were negative, and two further echocardiograms showed no changes.

After initial clinical improvement with piperacillin-tazobactam, the patient deteriorated, with dyspnoea, pyrexia, and lung crepitations. He was switched to ciprofloxacin and non-steroid anti-inflammatory drugs (NSAIDs), but C-reactive protein (CRP) remained elevated. Following a review by infectious disease, the patient was confirmed negative for HIV. His only previous TB contact was an acquaintance who had active TB 30 years previously. Polymerase chain reaction of the bronchoalveolar lavage and pleural aspirate was negative for TB. However, the interferon-gamma release assay (QuantiFERON) was positive for TB. At this point, the patient was afebrile and clinically well, but his CRP was 142 mg/dL. Though latent TB was considered a low-probability differential, the patient was started on a four-drug regimen.

**Figure 4:**
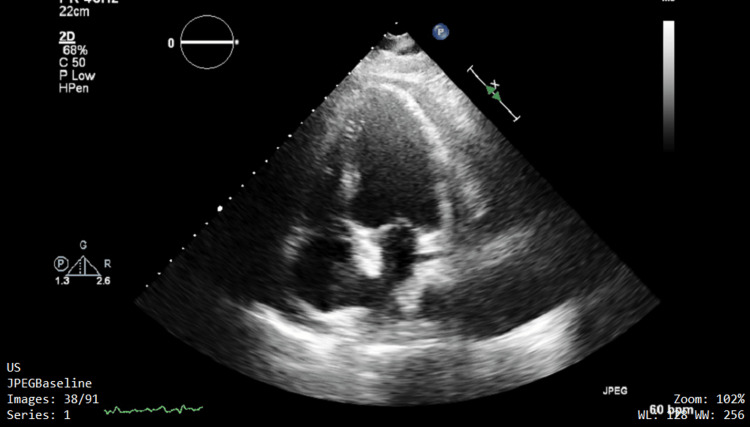
Previously noted pericardial fluid with an organized/fibrinous/thrombotic appearance Regional wall abnormalities and a septal bounce were noted here.

A repeat echocardiogram identified new regional wall motion abnormalities, new septal bounce, and an organized/fibrotic/thrombotic pericardial effusion with a thickened pericardium (Figure 4). Cardiology requested a cardiac MRI to clarify these echo findings and requested cardiothoracic surgery involvement as pericardiectomy was anticipated. Prednisolone was added to the four-drug regimen, as corticosteroids are indicated in pericardial TB. The cardiac MRI showed constrictive pericarditis, and it was felt that pericardiectomy was warranted as treatment regardless of the precise etiology.

The patient was transferred to a specialist center for pericardiectomy. The excised pericardium was positive for mycobacterium TB. Prednisolone was stopped, as it was felt that the source of the TB had been removed and was no longer indicated. The postoperative period was not without incident; the patient experienced a syncopal episode, MRI brain showed bilateral acute infarcts, and the patient was given therapeutic anticoagulation. A postoperative echocardiogram showed severely impaired left ventricular systolic function with an ejection fraction estimated at 40%, though with poor visualization. This new diagnosis of heart failure necessitated the addition of guideline-directed heart failure therapy, further adding to the patient's multipharmacy and comorbidity. The patient was readmitted to hospital two months later following heavy bleeding per rectum. This was attributed to excess anticoagulation due to drug interaction with four-drug therapy. The ethambutol and rifampicin/isoniazid/pyrazinamide combination were stopped and the rifampicin/isoniazid combination was used instead for TB therapy. At the cardiology outpatient review three months after pericardiectomy, the patient reported no new complaints.

## Discussion

The significance of this case is that it reinforces the notion of always considering tuberculosis. Even in the absence of risk factors, in a non-endemic area, without significant prior exposure, with multiple negative tests, TB remains a reasonable differential.

TB has been implicated as a cause of 4% of pericarditis cases [5]. Though extrapulmonary tuberculosis (EPTB) is now almost as common a diagnosis as pulmonary TB in the UK, pericardial TB accounts for less than 1% of EPTB [2].

Pericardial TB can be rapidly fatal if it presents acutely with cardiac tamponade caused by massive pericardial fluid. The treatment for this is urgent drainage with pericardiocentesis or pericardiectomy, and corticosteroids [2]. This patient had a more indolent disease progression, beginning with pericardial effusion without hemodynamic disruption and later developing into constrictive pericarditis, which prompted an urgent intervention. While TB had been confirmed via a Quantiferon test, the nidus of the infection was not confirmed until after pericardiectomy. Pericardial fluid has been used to diagnose pericardial TB [6], but this was not done in this instance, as the size of the effusion did not initially warrant pericardiocentesis and the pericardium was not felt to be the site of TB infection prior to the pericardiectomy.

In endemic regions, TB is the most common cause of pericardial effusion [7]. However, the TB differential was not considered highly probable in the absence of any risk factors or classic symptoms of TB. Though some ‘common symptoms of pericardial tuberculosis’ (Trautner & Darouiche, 2010) were present (cough and dyspnoea), these mostly resolved following antibiotic therapy, and only the patient’s raised CRP and residual pericardial effusion delayed discharge. Notably, the patient had no chest pain, night sweats, orthopnoea, weight loss, or ankle edema, which are all associated with pericardial TB.

Following the finding that the pleural effusion was exudative, TB was placed higher on the differential. However, given the patient’s history of prostate cancer, metastasis was considered the most likely cause. This reasoning was supported by multiple blood cultures and PCR being negative for TB, as were bronchoalveolar washings and sputum cultures. Negative investigations are not uncommon in pericardial TB, and this highlights the importance of pericardial tissue for accurate diagnosis [1].

Four stages of pericardial TB have been described [8]. The first stage is fibrinous exudation with early granuloma formation. The second stage is serosanguineous lymphocyte-predominant exudative effusion. Following this, the effusion becomes organized with granulomatous caseation and fibrin-induced pericardial thickening. The final stage is constrictive scarring, leading to constrictive pericarditis. This patient was likely initially in the second stage with serosanguineous effusion, then progressed through the third stage of pericardial thickening, and to the final stage of constrictive pericarditis.

A six-month course of anti-TB medication is recommended by Moyasi et al. in their 2005 review paper, extended to nine months in patients with diabetes. The addition of adjunctive steroids reduces mortality and hastens clinical recovery [1]. The risk of tamponade warranted pericardiectomy in this case, but aspiration of the effusion may be sufficient in the acute situation. Pericardiectomy was also justified in this patient to avoid the sequelae of constrictive pericarditis. Operative mortality is between 4% and 8% for pericardiectomy [9], and the perioperative morbidity seen in this patient may be expected following such a procedure, which should be short-term. When this patient was seen in an outpatient clinic two months after surgery, he reported no new complaints and was clinically and functionally well.

## Conclusions

In general, TB can mimic other, more common diseases. TB needs to be considered as a potential diagnosis in cases where there is uncertainty, multiple symptoms, or non-resolution with initial management. Even in non-endemic areas and in the absence of typical risk factors, TB is still present and should be considered in the differential.

Pericardial TB can be a difficult diagnosis to obtain. Its clinical presentation can vary from indolent non-specific infectious symptoms to acute cardiac tamponade. It is only with a multidisciplinary approach that the diagnosis and, ultimately, effective treatment can be achieved.
